# Cimigenoside Attenuates Ulcerative Colitis by Inhibiting Oxidative Stress and Inflammation via Sirtuin 3 Enhancement in Mice

**DOI:** 10.3390/antiox15040428

**Published:** 2026-03-28

**Authors:** Jie-Ming Chang, Yu-Mei Shan, Yu-Hang Zhou, Jing-Wen Lu, Hao Ding, Ying Zhou, Yu-Fan Ji, Rui-Jie Tao, Wen-Hao Zhu, Ting-Dong Yan, Zhao-Guo Liu

**Affiliations:** 1 Department of Pharmacology, School of Pharmacy, Nantong University, Nantong 226019, China; 2 Department of Clinical Medicine, Xinlin College, Nantong University, Nantong 226000, China

**Keywords:** cimigenoside, ulcerative colitis, inflammation, oxidative stress, sirtuin 3

## Abstract

Ulcerative colitis (UC) is a highly prevalent chronic non-specific intestinal inflammatory disorder for which effective therapeutic options are urgently needed. The active component cimigenoside (CIM) possesses promising anti-inflammatory bioactivity; however, its therapeutic efficacy and underlying molecular mechanism against UC remain to be fully elucidated. The present study aimed to investigate the effects and possible mechanisms of CIM on dextran sodium sulfate (DSS)-induced UC. Mice received drinking water containing 2.5% DSS to induce a UC model, and were then treated with different dosages of CIM for 10 consecutive days. The results found that CIM restored the colonic length, alleviated pathological damage to the colon, preserved intestinal mucosal barrier integrity, and inhibited colonic oxidative stress and inflammatory responses in DSS-induced mice. Additionally, DSS induction reduced the expression of sirtuin 3 (SIRT3) protein in the colonic tissues of mice; however, this was improved by treatment with CIM. Notably, the above protective roles of CIM on DSS-induced UC were unavailable in SIRT3-knockout (SIRT3-KO) mice. Notably, the docking score of CIM binding to SIRT3 is −11.3 kcal/mol, suggesting that CIM could directly bind to SIRT3. Collectively, CIM directly binds to SIRT3 and upregulates its protein expression, which in turn inhibits colonic inflammation and oxidative stress, thereby exerting anti-UC effects.

## 1. Introduction

Ulcerative colitis (UC) is a chronic inflammatory bowel disease that affects the colon, characterized by a relapsing-remitting course. Currently, there is no effective clinical prevention and treatment strategy for this condition [1]. Colonic inflammation and oxidative stress play pivotal roles in the pathogenesis of UC. Colonic inflammation has been recognized as the primary initiator of UC [2]. Under physiological conditions, the intestinal immune system accurately discriminates between foreign pathogens and beneficial microbiota, triggering inflammatory responses to eliminate pathogens while maintaining immune tolerance toward beneficial bacteria [3]. However, in UC patients, this immune homeostasis is disrupted and causes excessive and uncontrolled inflammatory cascades [4]. Sustained inflammation progressively impairs the intestinal mucosal barrier. Once this barrier is compromised, intestinal bacterial endotoxins and harmful metabolites gain easier access to the submucosal layer. This, in turn, further activates the immune system and amplifies inflammatory responses [5]. Thus, effective suppression of inflammatory responses with a subsequent improvement in the colonic inflammatory microenvironment represents a key therapeutic strategy for UC intervention.

Colonic oxidative stress is closely associated with UC pathogenesis [6]. During UC onset, colonic tissues generate excessive reactive oxygen species (ROS) that surpass the body’s inherent scavenging ability, leading to ROS accumulation and the development of oxidative stress [7]. To counteract UC-associated intestinal oxidative stress, the body employs an intrinsic defense mechanism centered on the antioxidant enzyme system. This system comprises key enzymes such as superoxide dismutase (SOD), glutathione peroxidase (GSH-Px), and catalase (CAT) [8]. These enzymes exert targeted ROS-scavenging effects, interrupting the chain reaction of oxidative damage and thereby preserving the balance between oxidative stress and antioxidant defense [9]. Consequently, enhancing the activity of these antioxidant enzymes with a subsequent reduction in oxidative stress serves as an effective approach to alleviate UC symptoms and pathology.

Sirtuins (SIRTs), a family of nicotinamide adenine dinucleotide (NAD^+^)-dependent histone deacetylases, are capable of regulating mitochondrial acetylation levels [10]. The SIRT family comprises seven members, designated SIRT1 through SIRT7. Among mitochondrial SIRTs, all exhibit deacetylase activity, with SIRT3 demonstrating significantly higher activity than SIRT4 and SIRT5. Specifically, SIRT3 plays a pivotal role in regulating the mitochondrial acetylome, and its deacetylase function is critical for modulating mitochondrial biological processes as well as various pathophysiological events, including the maintenance of redox homeostasis [11]. In the context of intestinal diseases, SIRT3 is involved in the pathogenesis of multiple intestinal disorders, including UC, sepsis-induced small intestinal injury, intestinal ischemia/reperfusion injury, intestinal barrier dysfunction, and radiation-induced intestinal damage [12,13,14]. Importantly, the effective enhancement of SIRT3 has been shown to alleviate the progression of these diseases [15,16]. Therefore, identifying agents that can enhance SIRT3 expression represents a promising therapeutic strategy for UC treatment.

Cimigenoside (CIM), a cycloartane-type triterpenoid compound extracted from *Cimicifuga dahurica* (Turcz.) Maxim., is one of the primary pharmacologically active components of this medicinal herb [17]. The chemical structure of CIM is shown in Section 3.1, and the molecular weight of CIM is 620.81 kDa. Previous research has confirmed that CIM possesses multiple biological activities, including anti-tumor, anti-inflammatory, immunomodulatory, and anti-apoptotic effects [18]; however, its role and regulatory mechanisms in UC remain unreported to date. The present study aims to investigate the effects and the detailed mechanism of CIM on UC: specifically, the role of SIRT3-mediated colonic inflammation regulation in the anti-UC effect of CIM will be clarified.

## 2. Materials and Methods

### 2.1. Source and Related Information of the Compound

Cimigenoside (CIM) (Cat No. IYT1771, purity ≥ 98%) was purchased from Beijing Solarbio Science & Technology Co., Ltd. CIM is a cycloartane-type triterpenoid. The product instructions are attached in the Supplementary Material.

### 2.2. Animal Treatments

(a) Establishment of a DSS-induced UC mouse model, using male C57BL/6 mice and CIM treatment.

A total of 50 male C57BL/6 mice (4 weeks, 18–20 g) were randomly divided into five groups with 10 mice in each group, and sulfasalazine (SASP) was selected as the positive control drug. The specific grouping was as follows: control group, DSS group, DSS + CIM (5 mg/kg) group, DSS + CIM (20 mg/kg) group, and DSS + SASP (200 mg/kg) group. The UC mouse model was established by allowing free access to drinking water containing 2.5% DSS (Bide Pharmatech Ltd., Shanghai, China) for 7 consecutive days, during which no normal drinking water was provided, and the DSS solution was replaced daily. Mice in the control group had free access to normal drinking water and food. Drug intervention was initiated on the 8th day after modeling: mice in the CIM treatment groups were given CIM (5 or 20 mg/kg, Beijing Solarbio Science & Technology Co., Ltd., Beijing, China) by gavage once a day for 10 consecutive days. Mice in the SASP treatment group were given SASP (200 mg/kg, Shanghai Yuanye Bio-Technology Co., Ltd., Shanghai, China) by gavage once a day for 10 consecutive days. Mice in the control group and DSS group were given an equal volume of normal saline by gavage once a day for 10 consecutive days. During the experiment, the body weight of each mouse was recorded daily, and the Disease Activity Index (DAI) score was calculated to evaluate the severity of colitis. At the end of the experiment, blood was collected from the mice via eyeball enucleation, serum was obtained by centrifugation, and mouse colon tissues were collected for subsequent analysis.

(b) Establishment of a DSS-induced UC mouse model, using male 129S1/SvImJ mice and SIRT3 knockout mice and CIM treatment.

Male 129S1/SvImJ mice (wild-type, WT) (4 weeks, 18–20 g) and SIRT3 knockout (SIRT3 KO) (4 weeks, 18–20 g) mice were randomly divided into six groups, with 10 mice in each group. The specific grouping was as follows: WT + control group, WT + DSS group, WT + DSS + CIM (20 mg/kg) group, KO + control group, KO + DSS group, and KO + DSS + CIM (20 mg/kg) group. Mice in the WT + DSS group, WT + DSS + CIM (20 mg/kg) group, KO + DSS group, and KO + DSS + CIM (20 mg/kg) group were used to establish UC mouse models by free access to drinking water containing 2.5% DSS for 7 consecutive days. During this period, no normal drinking water was provided, and the DSS solution was replaced daily. Mice in the WT + control group and KO + control group were given free access to normal drinking water and food. Drug intervention was initiated on the 8th day after modeling: mice in the WT + DSS + CIM (20 mg/kg) group and KO + DSS + CIM (20 mg/kg) group were given CIM by gavage once a day for 10 consecutive days. During the experiment, the body weight of each mouse was recorded daily, and the DAI score was calculated to evaluate the severity of the colitis. The procedures for collecting the serum and colon tissues were the same as described above.

### 2.3. Animal Care and Monitoring

All efforts were made to minimize animal suffering and distress. Animals were housed in groups on soft bedding in a controlled environment with enrichment items (nesting material, shelters). Food and water were provided ad libitum. Body weight and clinical signs were monitored daily. Isoflurane inhalation anesthesia was used for experiments to relieve animal pain. The experimental environment temperature and humidity were controlled to reduce animal stress. Humane endpoints: predefined humane endpoints were strictly followed to prevent severe suffering. Any animal meeting one or more of the following criteria was immediately euthanized by CO_2_ inhalation followed by cervical dislocation. Weight loss: Sustained weight loss exceeding 20% of the initial body weight within 48 h. Clinical signs: Severe, persistent diarrhea leading to dehydration (assessed by skin tenting), complete anorexia (no food intake for 24 h), or extreme lethargy (unresponsive to gentle stimulation). Physical condition: Signs of severe pain (e.g., hunched posture, vocalization upon palpation), self-mutilation, or any condition impairing the ability to access food or water. Monitoring: All animals were monitored at least twice daily (morning and evening) for their general condition, food/water intake, and clinical signs. Their body weight was recorded daily. During the expected peak of the disease (days 3–6), the monitoring frequency was increased to three times daily.

### 2.4. Histological Analysis

Male mice colon tissues were fixed in 4% paraformaldehyde for 24 h and sectioned into 5 μm thin slices. The sections were subjected to hematoxylin eosin (H&E) to observe the pathological damage of the mouse colon, and alcian blue (AB) staining to evaluate the expression of colonic mucin, as well as periodic acid-schiff (PAS) staining to assess the number of colonic goblet cells. Images were captured under an optical microscope.

### 2.5. Disease Activity Index (DAI) Score

The disease activity index score is calculated using three domains: weight loss, stool consistency, and presence of blood in the stool. The weight loss percentage is assessed on a 0–4 scale: 0 (none), 1 (1–5%), 2 (5–10%), 3 (10–20%), and 4 (>20%). The stool consistency is rated on a 0–4 scale: 0 (normal), 2 (soft), and 4 (loose). The presence of bleeding is scored on a 0–4 scale: 0 (absent), 2 (tinged), and 4 (present). The final index score is the average of these three domain scores.

### 2.6. ROS Detection

We collected male mice colon tissues and prepared the homogenates. We centrifuged the tissue homogenates at 3000× *g* and 4 °C for 15 min. In accordance with the manufacturer’s instructions, we determined the reactive oxygen species’ (ROS) concentration in the supernatant using the ROS probe dichlorofluorescin diacetate (DCFH-DA), at a dilution ratio of 1:1000. We measured the fluorescence intensity of DCFH-DA in each well with a SpectraMax i3x multimode microplate reader (Molecular Devices, LLC, San Jose, CA, USA).

### 2.7. Detection of Antioxidant Enzyme Activity

After harvesting the mouse colon tissues, they were immediately minced and ground on a pre-chilled ice plate to prepare tissue homogenates. The homogenates were then centrifuged at 12,000× *g* for 10 min at 4 °C. The supernatants were collected, and the activities of SOD (Grass Source Biotechnology Co., Ltd., Guangzhou, China) and GSH-Px (Shanghai ToYongBio Biotechnology Co., Ltd., Shanghai, China) were assayed following the manufacturer’s instructions, with the experiment performed in triplicate.

### 2.8. Myeloperoxidase (MPO) Activity Assay

The serum MPO activity was measured using an MPO detection kit (Shanghai Macklin Biochemical Co., Ltd., Shanghai, China), following these steps: Serum samples were mixed with reaction solution and incubated at 37 °C for 30 min. The mixture was then held at 60 °C for 10 min, after which 50 μL of hydrogen peroxide was added. Absorbance at 460 nm was determined using a multifunctional microplate reader. MPO activity was defined as 1 unit (U): the amount of enzyme required to degrade 1 μM hydrogen peroxide at 37 °C. Results were expressed as units of activity per liter of serum.

### 2.9. Western Blotting

The male mice colon tissues were minced and lysed in RIPA buffer (supplemented with protease and phosphatase inhibitors) to prepare the homogenates. The protein concentration was quantified using a BCA assay kit (Beyotime, Shanghai, China). Proteins (50 μg per well) were separated by SDS-PAGE and transferred to PVDF membranes. Post-transfer, membranes were blocked with 5% non-fat milk for 2 h, then incubated with primary antibodies against anti-SIRT3 (1:1000, Cell Signaling Technology, Danvers, MA, USA); anti- Occludin (1:2000, Affinity, Affinity Biosciences Co., Ltd., Cincinnati, OH, USA); anti-Nrf2 (1:2000, Proteintech, Rosemont, IL, USA); anti-Lamin B1 (1:1000, Cell Signaling Technology, Danvers, MA, USA); anti-β-Tubulin (1:2000, Engibody Biotechnology, Dover, DE, USA); and anti-GAPDH (1:1000, Proteintech, Rosemont, IL, USA) at 4 °C overnight. The next day, the primary antibodies were recovered, and the membranes were washed 5 times with TBST prior to incubation with secondary antibodies for 1.5 h. After an additional 5 TBST washes, bands were visualized using ECL luminescent solution.

### 2.10. Quantitaive Real-Time PCR

The total RNA was extracted from the colon tissues of male mice following the instructions provided with the TRIzol reagent kit (Invitrogen, Carlsbad, CA, USA). Subsequently, the total RNA was reverse-transcribed into complementary DNA (cDNA), in accordance with the protocols of the reverse transcription kit. Using the prepared cDNA as a template, a real-time fluorescence quantitative polymerase chain reaction (qPCR) was then performed with the SYBR Green reagent. The expression level of the target gene was normalized via the 2^−ΔΔCT^ method, with GAPDH expression serving as the internal reference. The primer sequences (Sangon Biotech Co., Ltd., Shanghai, China) are listed as follows: mice *SIRT1*, sense 5′-TTGTGAAGCTGTTCGTGGAG-3′ and antisense 5′-GGCGTGGAGGTTTTTCAGAT-3′; mice *SIRT2*, sense 5′-AGCCAACCATCTGCCACTAC-3′ and antisense 5′-CCAGCCCATCGTGTATTCTT-3′; mice *SIRT3*, sense 5′-GAGCGGCCTCTACAGCAAC-3′ and antisense 5′-GGAAGTAGTGAGTGACATTGGG-3′; mice *SIRT4*, sense 5′-GAGCAACTGGGAGAGACTGG-3′ and antisense 5′-ACAGCACGGGACCTGAAA-3′; mice *SIRT5*, sense 5′-CGCTGGAGGTTACTGGAGA-3′ and antisense 5′-CGTCAATGTTCTGGGTGATG-3′; mice *SIRT6*, sense 5′-CATGGGCTTCCTCAGCTTC-3′ and antisense 5′-AACGAGTCCTCCCAGTCCA-3′; mice *SIRT7*, sense 5′-GGACAGAGCACCAATACCG-3′ and antisense 5′-GGACAGAGCACCAATACCG-3′; mice *IL-1β*, sense 5′-GCAACTGTTCCTGAACTCAACT-3′ and antisense 5′-ATCTTTTGGGGTCCGTCAACT-3′; mice *IL-6*, sense 5′-CCCCAATTTCCAATGCTCTCC-3′ and antisense 5′-CGCACTAGGTTTGCCGAGTA-3′; mice *TNF-α*, sense 5′-ATGTCTCAGCCTCTTCTCATTCCT-3′ and antisense 5′-GGGTCTGGGCCATAGAACTGA-3′; mice *ZO-1*, sense 5′-GTTGGTACGGTGCCCTGAAAGA-3′ and antisense 5′-GCTGACAGGTAGGACAGACGAT-3′; mice *MUC2*, sense 5′-CATTTACAGCCTTACTGGGAGG-3′ and antisense 5′-ATGCAGTCAAATCTGGTGGCA-3′; mice *Occludin*, sense 5′-TGGCAAGCGATCATACCCAGAG-3′ and antisense 5′-CTGCCTGAAGTCATCCACACTC-3′; mice *Claudin-1*, sense 5′-GGACTGTGGATGTCCTGCGTTT-3′ and antisense 5′-GCCAATTACCATCAAGGCTCGG-3′; mice *COX-2*, sense 5′-TTTGCCCAGCACTTCACCCAT-3′ and antisense 5′-AAGTGGTAACCGCTCAGGTGT-3′; mice *iNOS*, sense 5′-CTGCAGCACTTGGATCAGAACCTG-3′ and antisense 5′-GGGAGTAGCCTGTGTGCACCTGGAA-3′; mice *NQO1*, sense 5′-CAGCCAATCAGCGTTCGGTA-3′ and antisense 5′-CTTCATGGCGTAGTTGAATGATGTC-3′; and mice *GAPDH*, sense 5′-CCTCGTCCCGTAGACAAAATG-3′ and antisense 5′-TGAGGTCAATGAAGG GGTCGT-3′.

### 2.11. Enzyme-Linked Immunosorbent Assay (ELISA)

For tissues: The male mice colons were harvested, minced, and homogenized with lysis buffer. The homogenates were centrifuged, and the supernatants were collected. The levels of inflammatory factors in the tissues were determined using ELISA kits (NeoBioscience Technology Co., Ltd., Shenzhen, China), following the manufacturer’s instructions.

For serum: The male mice blood was centrifuged (1000× *g*, 4 °C, 10 min) to obtain serum. The levels of TNF-α, IL-1β, IL-6, IFN-γ, and IL-10 in serum were measured using ELISA kits, according to the manufacturer’s protocols. The optical density (OD value) was read at 450 nm using a microplate reader.

### 2.12. Immunohistochemical Staining (IHC)

Paraformaldehyde-fixed, paraffin-embedded tissue sections were subjected to an immune histochemistry (IHC) assay. Briefly, slides were deparaffinized and rehydrated through graded ethanol solutions and distilled water, then immersed in 3% H_2_O_2_ in methanol for 30 min. After PBS washes, they were incubated in 10% normal goat serum for 30 min, followed by additional washes. Overnight incubation at 4 °C was performed with primary antibodies: SIRT3 (1:500, Proteintech, Rosemont, IL, USA). Subsequent steps included three PBS washes, incubation with secondary antibodies, another PBS wash, development using a DAB commercial kit (CoWin Biosciences, Taizhou, China), counterstaining with hematoxylin, and a final assessment of protein expression via photography under a light microscope at 200× magnification.

### 2.13. Measurement of SIRT3 Activity

SIRT3 deacetylase activity was measured using the CycLex SIRT3 Deacetylase Fluorometric Assay Kit (Cat. No. CY-1153V2, MBL International Co., Ltd., Nagoya, Japan), a specialized fluorometric kit designed for the quantitative detection of SIRT3 deacetylase enzymatic activity. Briefly, fresh colon tissue was rinsed with ice-cold PBS to remove impurities, weighed, and homogenized on ice in a kit lysis buffer containing 1% protease inhibitor cocktail. After 30 min of lysis on ice, homogenates were centrifuged at 12,000× *g* for 15 min at 4 °C, and supernatants were collected. Total protein concentration was quantified by a BCA assay, and samples were diluted to 1–2 mg/mL. Reactions were performed in opaque black 96-well plates with a 50 μL total volume: 20 μL diluted protein supernatant was mixed with 30 μL kit master mix (assay buffer, fluorogenic substrate, and NAD^+^). Plates were sealed and incubated at 37 °C for 60 min, and fluorescence intensity was detected at an excitation of 350 nm and an emission of 450 nm. Activity was presented as a relative value compared with that of the control group. All samples were assayed in triplicate.

### 2.14. Molecular Docking

The molecular docking of CIM with proteins was preformed using AutoDockTools-1.5.7 (https://ccsb.scripps.edu/mgltools/downloads/, accessed on 16 March 2026). The negative grid score showed the combination of CIM with proteins.

### 2.15. Evaluation of Systemic Toxicity

Two experimental groups were established using male C57BL/6 mice (4 weeks, 18–20 g): a control group and a high-dose CIM-alone administration group. Mice in the CIM-alone treated group were given CIM (20 mg/kg) by gavage once a day for 10 consecutive days, while mice in the control group were given normal saline by gavage once a day for 10 consecutive days. At the end of the experiment, blood was collected from the mice via eyeball enucleation, the serum was obtained by centrifugation for ELISA as described in Section 2.11, and heart, liver, spleen, lung and kidney tissues were collected for H&E analysis, as described in Section 2.4.

### 2.16. Statistical Analysis

All data are expressed as mean ± standard deviation (mean ± SD), and analysis was performed by one-way analysis of variance and a Bonferroni post hoc test. Statistical comparisons were also performed between the low-dose and high-dose test compound groups versus the positive control group, respectively. Values of *p* less than 0.05 are considered to be statistically significant.

## 3. Results

### 3.1. CIM Alleviated DSS-Induced UC in Mice

**Figure 1:**
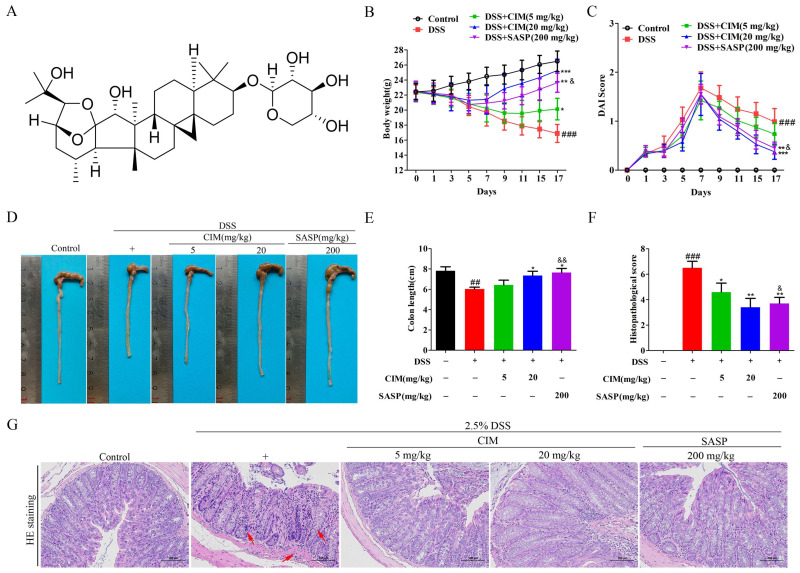
CIM-attenuated UC in DSS-induced male C57BL/6 mice. (**A**) The chemical structure of CIM was drawn by the authors using ChemDraw 23.0 software (Revvity Signals, Waltham, MA, USA), according to its confirmed structure. (**B**) Body weight. (**C**) DAI score. (**D**) Representative images of the colon. (**E**) Colon length. (**F**) Histopathological score. (**G**) Representative images of colon tissue with HE staining, Bar = 100 μm. Data are expressed as mean ± SD (*n* = 10). ^##^ *p* < 0.01, ^###^ *p* < 0.001 versus control; * *p* < 0.05, ** *p* < 0.01, *** *p* < 0.001 versus DSS; ^&^ *p* < 0.05, ^&&^ *p* < 0.01 versus CIM (5 mg/kg).

To investigate whether CIM exerts an anti-UC effect in vivo, a mouse model of UC was established by providing free access to drinking water supplemented with 2.5% DSS. The mice were subsequently administered CIM or SASP via gavage. The results showed that DSS induction reduced mouse body weight and elevated the DAI score, relative to the control group (Figure 1B,C), while CIM treatment effectively increased body weight and decreased the DAI score in mice (Figure 1B,C). Additionally, DSS induction shortened the colonic length in mice compared with the control group, and this reduction was reversed by treatment with CIM (Figure 1D,E). HE staining showed that DSS induction led to disorganized colonic tissue architecture and extensive inflammatory cell infiltration in mice, relative to the control group. Notably, CIM treatment effectively mitigated these pathological lesions in the colonic tissue (Figure 1F,G). Notably, CIM at 20 mg/kg exerted a comparable effect to that of the positive control on body weight, colonic length, and DAI (*p* > 0.05), whereas CIM at 5 mg/kg showed a significantly weaker effect than the positive control (*p* < 0.05) (Figure 1B–F). Collectively, CIM ameliorated DSS-induced UC in mice.

### 3.2. CIM Did Not Induce Systemic Toxicity in Mice

**Figure 2:**
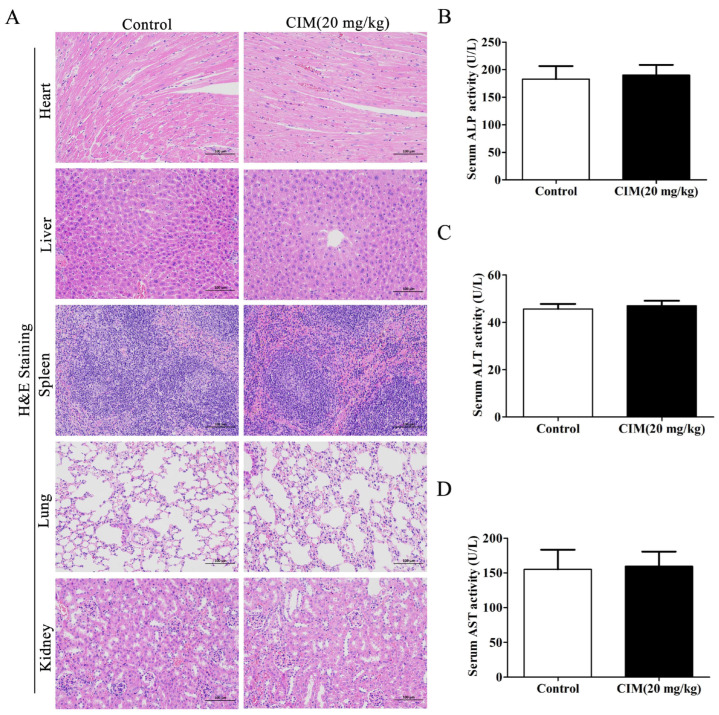
CIM did not induce systemic toxicity in male C57BL/6 mice. (**A**) Representative images of heart, liver, spleen, lung and kidney tissues with HE staining, Bar = 100 μm. (**B**) ALP in the serum. (**C**) ALT in the serum. (**D**) AST in the serum. Data are expressed as mean ± SD (*n* = 10).

We further investigated whether CIM elicits systemic toxicity in mice while exerting its anti-UC effects. To address this question, the pathological changes in the heart, liver, spleen, lung, and kidney between normal control mice and those treated with CIM (20 mg/kg) alone were compared. HE staining revealed no significant pathological abnormalities in the heart, liver, spleen, lung, or kidney of mice in the CIM monotherapy group (20 mg/kg), relative to the control group (Figure 2A). Furthermore, a comparative analysis of serological indices between the two groups showed no significant differences in the serum levels of ALP, ALT, and AST in the CIM-treated mice (20 mg/kg) compared with the control group (Figure 2B–D). Altogether, these results indicated that CIM did not induce systemic toxicity in mice.

### 3.3. CIM Attenuated Intestinal Mucosal Damage in DSS-Induced UC Mice

**Figure 3:**
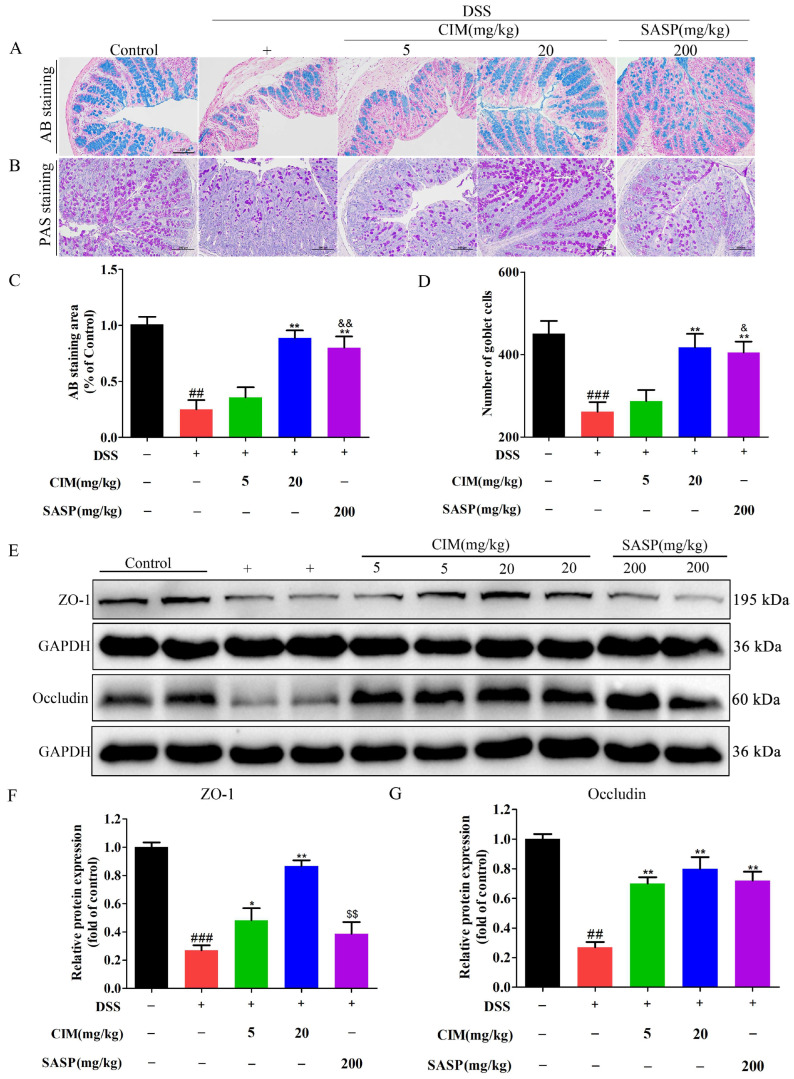
CIM-attenuated intestinal mucosal damage in DSS-induced male UC mice. (**A**) Representative images of mucin with alcian blue (AB) staining, Bar = 100 μm. (**B**) Representative images of goblet cells with periodic acid-schiff (PAS) staining, Bar = 100 μm. (**C**) AB staining areas were quantified. (**D**) Number of goblet cells were quantified. (**E**) Expression of ZO-1 and Occludin protein in mouse colonic tissues. (**F**) The expression of ZO-1 protein was quantified. (**G**) The expression of Occludin protein was quantified. Data are expressed as mean ± SD (*n* = 3 of 10 mice). ^##^ *p* < 0.01, ^###^ *p* < 0.001 versus control; * *p* < 0.05, ** *p* < 0.01 versus DSS; ^&^ *p* < 0.05, ^&&^ *p* < 0.01 versus CIM (5 mg/kg); and ^$$^ *p* < 0.01 versus CIM (20 mg/kg).

Mice with UC are frequently associated with intestinal mucosal damage [19]. Given this, we further investigated whether CIM exerts a protective effect against such damage. The results showed that DSS induction reduced mucin expression (Figure 3A,C) and decreased the number of goblet cells (Figure 3B,D) in colonic tissue, while CIM treatment increased both colonic mucin levels and goblet cell counts (Figure 3A–D). Notably, CIM at 20 mg/kg exhibited an effect that was comparable to that of the positive control in terms of colonic mucin levels and goblet cell counts (*p* > 0.05), while the effect of CIM at 5 mg/kg was weaker than that of the positive control (*p* < 0.05). Additionally, the effect of CIM on the protein expression of ZO-1 and Occludin, two key proteins that form tight junctions in the colonic mucosal epithelium, were examined by Western blot. Compared to the control group, DSS induction decreased the expression of ZO-1 and Occludin in mouse colonic tissue, whereas CIM treatment significantly increased their expression (Figure 3E–G). The positive control showed no obvious effect on the expression of ZO-1 (*p* > 0.05), but markedly increased the expression of Occludin (*p* < 0.01) (Figure 3F,G). Furthermore, CIM at 20 mg/kg exhibited a significantly stronger effect than the positive control on ZO-1 expression (*p* < 0.01) (Figure 3F), but not on Occludin expression (*p* > 0.05) (Figure 3G). In conclusion, CIM protects the intestinal mucosal barrier by alleviating DSS-induced intestinal mucosal damage in UC mice.

### 3.4. CIM Reduced DSS-Induced Inflammation in Both Serum and Colonic Tissues of Mice

**Figure 4:**
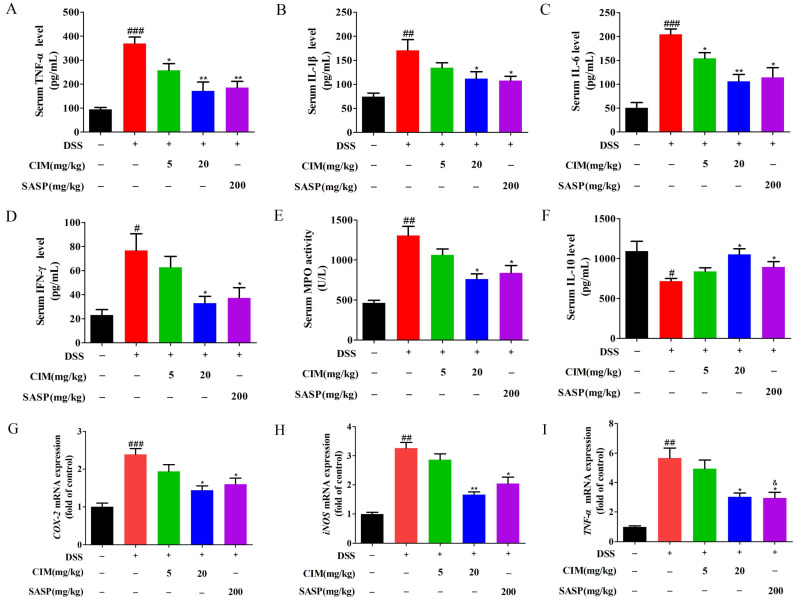
CIM reduced DSS-induced inflammation in serum and colonic tissues of male mice. (**A**) TNF-α in the serum. (**B**) IL-1β in the serum. (**C**) IL-6 in the serum. (**D**) IFN-γ in the serum. (**E**) MPO in the serum. (**F**) IL-10 in the serum. (**G**) *COX-2* mRNA expression in mouse colonic tissues. (**H**) *iNOS* mRNA expression in mouse colonic tissues. (**I**) *TNF-α* mRNA expression in mouse colonic tissues. Data are expressed as mean ± SD (*n* = 3 of 10 mice). ^#^ *p* < 0.05, ^##^ *p* < 0.01, ^###^ *p* < 0.001 versus control; * *p* < 0.05, ** *p* < 0.01 versus DSS; and ^&^ *p* < 0.05 versus CIM (5 mg/kg).

Accumulating evidence has demonstrated that intestinal tissues of mice with UC are persistently exposed to an inflammatory microenvironment [20]. Given this pathological feature, we hypothesized that CIM may exert inhibitory effects on colonic inflammation and thereby ameliorate the inflammatory microenvironment within the colonic tissues of UC mice. To validate this hypothesis, we further evaluated the impacts of CIM on the serum levels of multiple inflammatory mediators in UC mice. Compared with the control group, DSS induction elevated the serum levels of pro-inflammatory cytokines, including tumor necrosis factor-α (TNF-α), interleukin-1β (IL-1β), IL-6, and interferon-γ (IFN-γ) (Figure 4A–D), and enhanced serum myeloperoxidase (MPO) activity (Figure 4E) while reducing the serum level of the anti-inflammatory cytokine IL-10 (Figure 4F). In contrast, the administration of CIM effectively reduced the levels of pro-inflammatory cytokines, restored the serum IL-10 level, and attenuated MPO activity in UC mice (Figure 4A–F). In addition, the RT-PCR results showed that DSS induction increased the mRNA expression of *COX-2*, *iNOS* and *TNF-α* in the colonic tissues of mice, while treatment with CIM reduced the mRNA expression of the aforementioned proteins (Figure 4G–I). Notably, CIM at 20 mg/kg produced effects that were comparable to those of the positive control on mRNA level of *TNF-α* (*p* > 0.05). In contrast, CIM at 5 mg/kg showed significantly weaker effects than the positive control on the mRNA level of *TNF-α* (*p* < 0.05). Collectively, these results confirm that CIM can suppress systemic inflammatory responses and improve the colonic inflammatory microenvironment in DSS-induced UC mice.

### 3.5. CIM Suppressed DSS-Induced Oxidative Stress in Mouse Colonic Tissues

**Figure 5:**
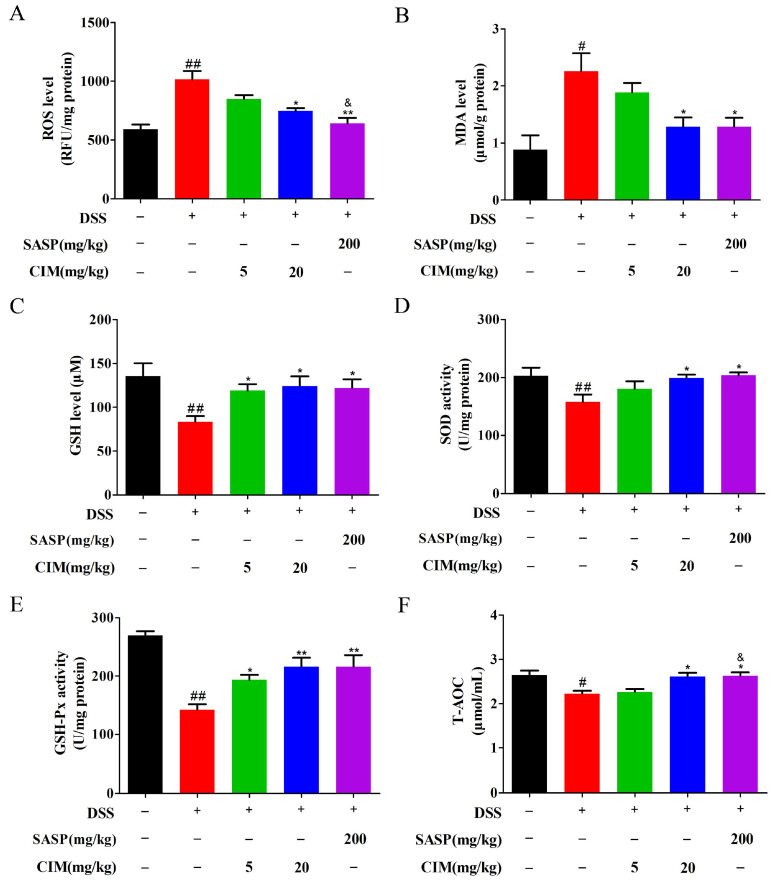
CIM suppressed DSS-induced oxidative stress in male mice colonic tissues. (**A**) ROS level in the mouse colonic tissues. (**B**) MDA content in the mouse colonic tissues. (**C**) GSH content in the mouse colonic tissues. (**D**) SOD activity in the mouse colonic tissues. (**E**) GSH-Px activity in the mouse colonic tissues. (**F**) T-AOC level in the mouse colonic tissues. Data are expressed as mean ± SD (*n* = 3 of 10 mice). ^#^ *p* < 0.05, ^##^ *p* < 0.01 versus control; * *p* < 0.05, ** *p* < 0.01 versus DSS; and ^&^ *p* < 0.05 versus CIM (5 mg/kg).

Compelling evidence has shown that DSS induction could also elicit robust oxidative stress responses in colonic tissues during UC pathogenesis. Therefore, we further examine the effects of CIM on DSS-induced oxidative stress in the colonic tissues of UC mice. Compared to those in the control group, DSS exposure elevated the levels of reactive oxygen species (ROS) and malondialdehyde (MDA) (Figure 5A,B), reduced glutathione (GSH) content (Figure 5C), and diminished the enzymatic activities of superoxide dismutase (SOD) and glutathione peroxidase (GSH-Px) (Figure 5D,E) together with the decreased total antioxidant capacity (T-AOC) levels (Figure 5F) in colonic tissues. Treatment with CIM effectively mitigated ROS and MDA accumulation, restored GSH content, enhanced the catalytic activities of SOD and GSH-Px, and elevated T-AOC levels in the colonic tissues of DSS-challenged UC mice (Figure 5A–F). Notably, CIM at 20 mg/kg produced effects that were comparable to those of the positive control on the ROS levels, MDA levels, GSH levels, SOD activity, GSH-Px activity, and T-AOC levels (*p* > 0.05). In contrast, CIM at 5 mg/kg showed significantly weaker effects than the positive control on ROS levels and T-AOC levels (*p* < 0.05). Collectively, these findings indicated that CIM suppressed DSS-induced oxidative stress in the colonic tissues of UC mice.

### 3.6. CIM Upregulated SIRT3 Expression Through Direct Binding to SIRT3 in Colonic Tissues of DSS-Induced UC Mice

**Figure 6:**
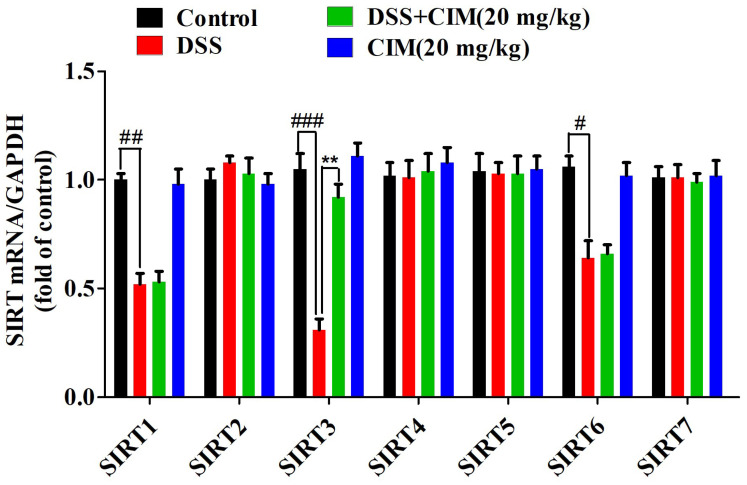
CIM specifically increased the mRNA expression of sirtuin 3 (SIRT3) in DSS-induced male UC mice. mRNA expression of SIRT1, SIRT2, SIRT3, SIRT4, SIRT5, SIRT6 and SIRT7 in mouse colonic tissues. Data are expressed as mean ± SD (*n* = 3 of 10 mice). ^#^ *p* < 0.05, ^##^ *p* < 0.01, ^###^ *p* < 0.001 versus control; and ^**^ *p* < 0.01 versus DSS.

**Figure 7:**
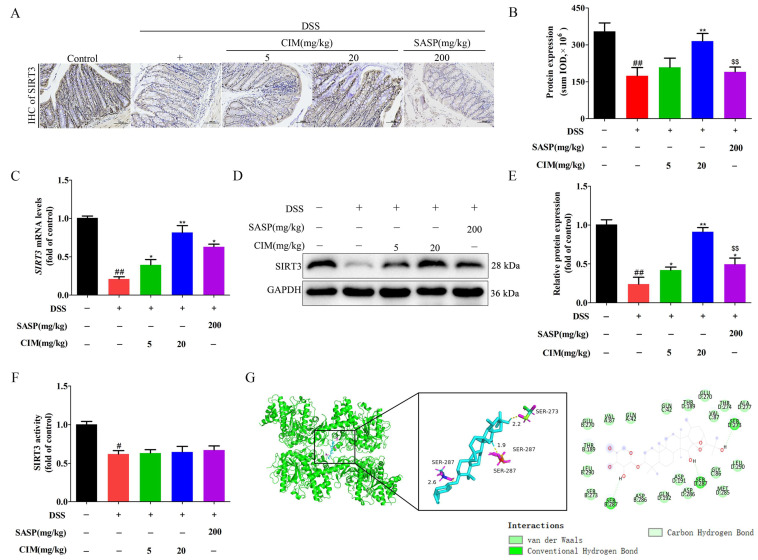
CIM increased SIRT3 expression in colonic tissues of DSS-induced male UC mice. (**A**) The representative images of colon tissues with immunohistochemical staining, Bar = 100 μm. (**B**) The expression of SIRT3 protein was quantified. (**C**) Expression of *SIRT3* mRNA in the mouse colonic tissues. (**D**) Expression of SIRT3 protein in mouse colonic tissues. (**E**) The expression of SIRT3 protein was quantified. (**F**) SIRT3 activity in the mouse colonic tissues. (**G**) Molecular docking simulation of CIM with mouse SIRT3. Data are expressed as mean ± SD (*n* = 3 of 10 mice). ^#^ *p* < 0.05, ^##^ *p* < 0.01 versus control; * *p* < 0.05, ** *p* < 0.01 versus DSS; and ^$$^ *p* < 0.01 versus CIM (20 mg/kg).

Accumulating evidence has demonstrated that multiple members of the sirtuin (SIRT) family are intricately implicated in the pathogenesis of UC [21]. This evolutionarily conserved family consists of seven isoforms: namely, SIRT1 through SIRT7. To elucidate whether CIM modulates the expression of SIRT family members in the context of UC, we assessed the effects of CIM on the mRNA levels of all seven SIRT isoforms in mouse colonic tissues using RT-PCR. Compared with the control group, DSS induction significantly decreased the mRNA expression of *SIRT1*, *SIRT3*, and *SIRT6* in the colonic tissues of mice. Notably, CIM administration specifically increased *SIRT3* mRNA expression (Figure 6), whereas it failed to exert regulatory effects on the mRNA levels of *SIRT1* or *SIRT6* in the colonic tissues of UC mice. Based on these results, we further investigated the effect of CIM on SIRT3 protein expression in the colon tissues of DSS-induced mice. Compared to the control group, immunohistochemical (IHC) analysis revealed that DSS induction downregulated SIRT3 protein expression in colon tissues, while CIM treatment upregulated SIRT3 protein expression (Figure 7A,B). However, IHC showed that SASP (positive control) did not significantly alter SIRT3 protein expression (Figure 7A,B). In addition, RT-PCR results showed that DSS induction decreased *SIRT3* mRNA expression in colon tissues compared with the control group, whereas CIM treatment increased *SIRT3* mRNA expression in colon tissues (Figure 7C). The Western blot results were consistent with the immunohistochemical results (Figure 7D,E). Notably, both the RT-PCR and Western blot showed that SASP could increase the mRNA and protein expression of SIRT3 in colon tissues. Furthermore, CIM at 20 mg/kg exhibited a significantly stronger effect than the positive control on the protein expression of SIRT3 (*p* < 0.01) (Figure 7D,E) but not on the mRNA expression of *SIRT3* (*p* > 0.05) (Figure 7C). Compared with the control group, the DSS challenge reduced SIRT3 activity in the mouse colon tissue, while CIM administration did not increase or restore it, suggesting no obvious effect of CIM on SIRT3 enzyme activity (Figure 7F). In addition, molecular docking results showed that the binding energy between CIM and SIRT3 is -11.3 kcal/mol, forming three hydrogen bonds with amino acid residues in the protein (Figure 7G), suggesting that CIM could directly bind to SIRT3. In conclusion, CIM upregulates SIRT3 expression by directly binding to SIRT3 in the colonic tissues of DSS-induced UC mice.

### 3.7. SIRT3 Knockout Attenuated the Anti-UC Effect of CIM in Mice

**Figure 8:**
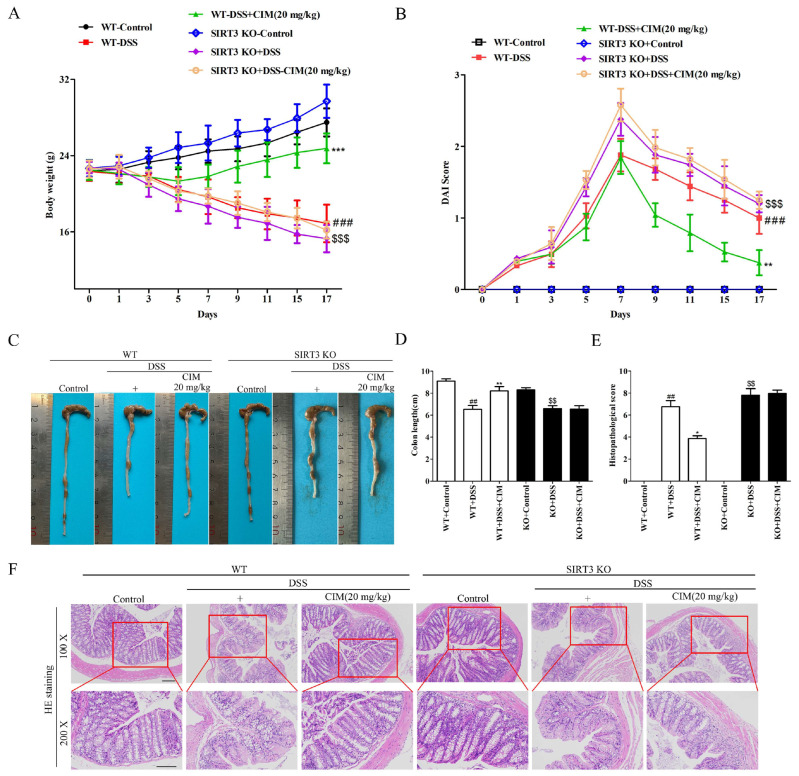
SIRT3 knockout attenuated the anti-UC effect of CI. (**A**) Body weight. (**B**) DAI score. (**C**) Representative images of the colon. (**D**) Colon length. (**E**) Histopathological score. (**F**) Representative images of colon tissue with HE staining, Bar = 100 μm. Data are expressed as mean ± SD (*n* = 10). ^##^ *p* < 0.01, ^###^ *p* < 0.001 versus WT-control; * *p* < 0.05, ** *p* < 0.01, *** *p* < 0.001 versus WT-DSS; and ^$$^ *p* < 0.01, ^$$$^ *p* < 0.001 versus SIRT3 KO-control.

The aforementioned findings indicated that CIM exerted a protective effect against DSS-induced UC and could increase SIRT3 protein expression in mouse colon tissues. Therefore, did the SIRT3 protein mediate the anti-UC effect of CIM? To address this question, SIRT3 knockout (SIRT3 KO) mice were used to investigate the impact of SIRT3 deletion on the anti-UC activity of CIM. The results showed that compared with the control group, DSS induction reduced the body weight of mice in both the wild-type (WT) group and the SIRT3 KO group. CIM treatment increased the body weight of WT mice but had no significant effect on the body weight of SIRT3 KO mice (Figure 8A). In addition, DSS induction elevated the DAI scores in both WT and SIRT3 KO mice, compared with the control group; however, the CIM treatment decreased the DAI score of WT mice but did not affect that of SIRT3 KO mice (Figure 8B). Additionally, DSS induction shortened the colon length in both WT and SIRT3 KO mice, and CIM treatment increased the colon length of WT mice but had no effect on that of SIRT3 KO mice (Figure 8C,D). Furthermore, the HE staining results revealed that DSS induction caused pathological damage to the colon tissues of both WT and SIRT3 KO mice. CIM treatment alleviated the pathological damage in the colon tissues of WT mice, but had no obvious protective effect on SIRT3 KO mice (Figure 8E,F). Taken together, SIRT3 knockout attenuated the protective effect of CIM against DSS-induced UC.

### 3.8. SIRT3 Knockout Attenuated the Protective Effect of CIM Against DSS-Induced Colonic Mucosal Injury

**Figure 9:**
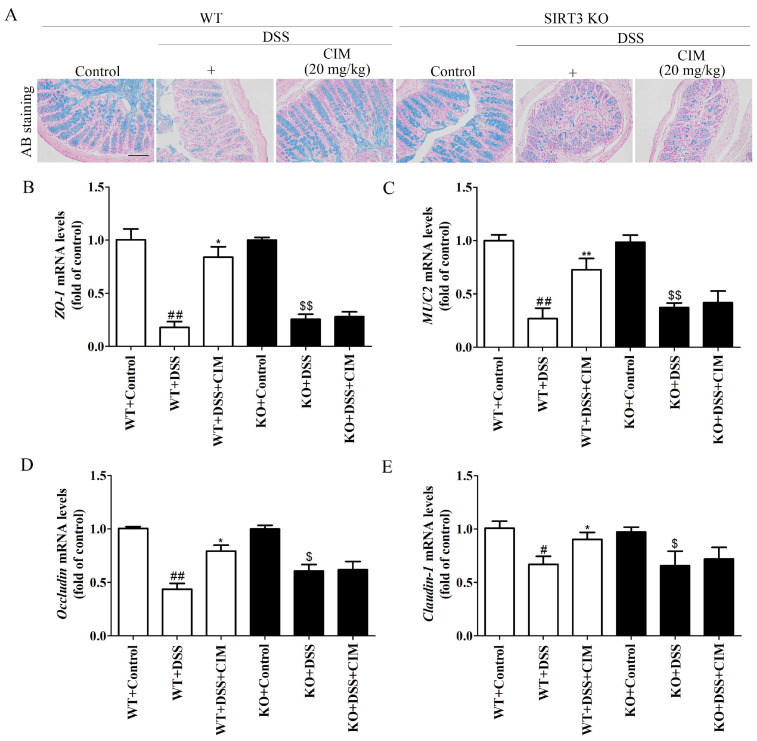
SIRT3 knockout attenuated the protective effect of CIM against DSS-induced colonic mucosal injury. (**A**) Representative images of mucin with alcian blue (AB) staining, Bar = 100 μm. (**B**–**E**) Expression of *ZO-1*, *MUC2*, *Occludin*, and *Claudin-1* mRNA in the mouse colonic tissues. Data are expressed as mean ± SD (*n* = 3 of 10 mice). ^#^ *p* < 0.05, ^##^ *p* < 0.01 versus WT-control; * *p* < 0.05, ** *p* < 0.01 versus WT-DSS; ^$^ *p* < 0.05, and ^$$^ *p* < 0.01 versus SIRT3 KO-control.

A further investigation was conducted to explore the effect of SIRT3 KO on the protective role of CIM in the colonic mucosal barrier. The alcian blue staining results demonstrated that DSS induction reduced the mucin levels in colonic tissues of both WT and SIRT3 KO mice. CIM treatment effectively elevated colonic mucin levels in WT mice, whereas no significant effect was observed in SIRT3 KO mice (Figure 9A). RT-PCR analysis revealed that DSS induction decreased the mRNA expressions of *ZO-1*, *MUC2*, *Occludin*, and *Claudin-1* in the colonic tissues of both WT and SIRT3 KO mice. In contrast, CIM treatment increased the mRNA expressions of these tight junction and mucin-related proteins in WT mice, but failed to exert a significant regulatory effect in SIRT3 KO mice (Figure 9B–D). Collectively, these findings indicated that SIRT3 KO attenuated the protective effect of CIM on the intestinal mucosal barrier in mice with UC.

### 3.9. SIRT3 Knockout Weakened the Anti-Colitis Effect of CIM in UC Mice

**Figure 10:**
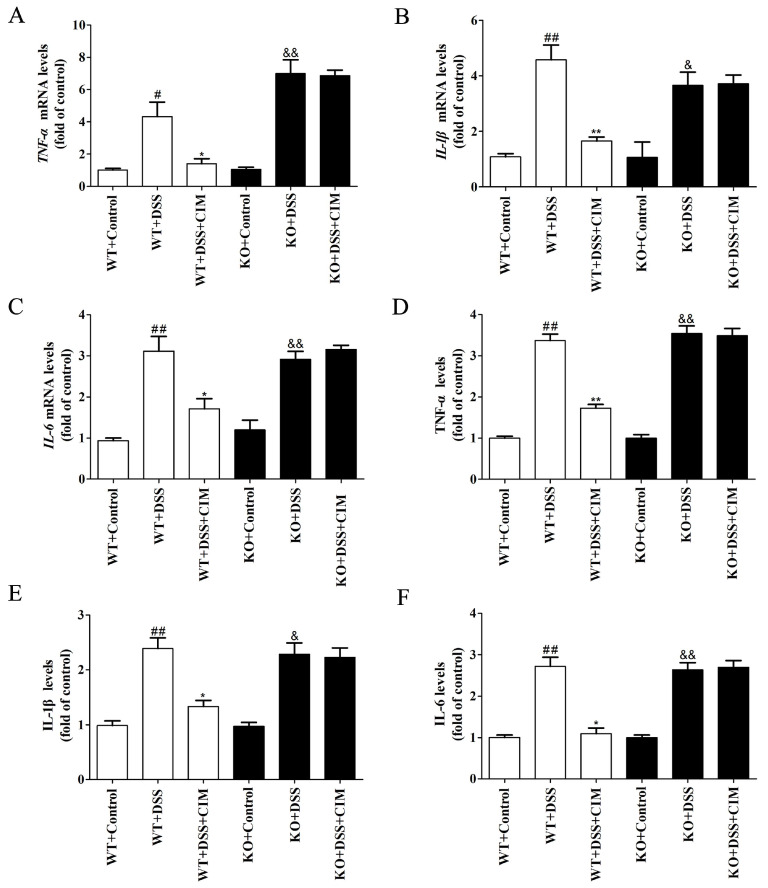
SIRT3 knockout weakened the anti-colitis effect of CIM in UC mice. (**A**–**C**) Expression of *TNF-α*, *IL-1β* and *IL-6* mRNA in the mouse colonic tissues. (**D**) TNF-α in the mouse colonic tissues. (**E**) IL-1β in the mouse colonic tissues. (**F**) IL-6 in the mouse colonic tissues. Data are expressed as mean ± SD (*n* = 3 of 10 mice). ^#^ *p* < 0.05, ^##^ *p* < 0.01 versus WT-control; * *p* < 0.05, ** *p* < 0.01 versus WT-DSS; ^&^ *p* < 0.05, and ^&&^ *p* < 0.01 versus SIRT3 KO-control.

Does SIRT3 protein also mediate the regulatory effect of CIM on colonic inflammation in ulcerative colitis (UC) mice? To address this question, further experiments were conducted to evaluate the impact of SIRT3 KO on the anti-colitic activity of CIM in UC mice. As illustrated in Figure 10A–C, DSS induction upregulated the mRNA expressions of *TNF-α*, *IL-1β*, and *IL-6*. Notably, CIM treatment effectively reduced the mRNA expressions of these inflammation-related mediators in WT mice, whereas no significant alterations were observed in SIRT3 KO mice. Additionally, DSS induction elevated the levels of TNF-α, IL-1β and IL-6 in the colonic tissues of both WT and SIRT3 KO mice; however, CIM treatment remarkably reduced the levels of these pro-inflammatory cytokines in the colonic tissues of WT mice, while exerting no significant influence on their levels in SIRT3 KO mice (Figure 10D–F). Altogether, these data indicated that SIRT3 knockout weakened the anti-colitic effect of CIM in mice with UC.

### 3.10. SIRT3 Knockout Abrogated the Antioxidative Stress Effect of CIM in the Colonic Tissues of UC Mice

**Figure 11:**
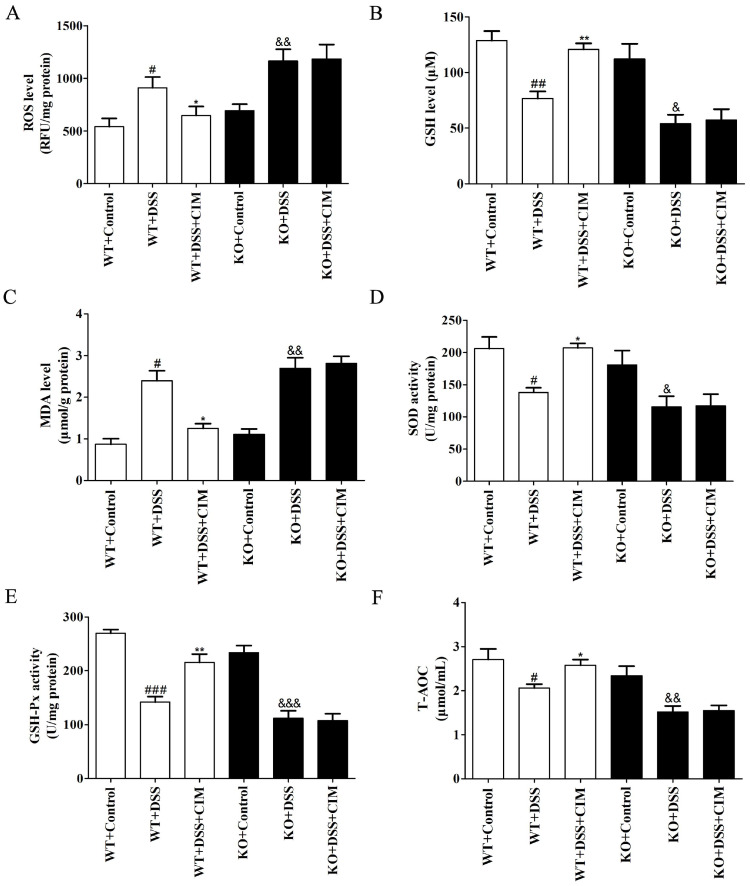
SIRT3 knockout abrogated the antioxidative stress effect of CIM in the colonic tissues of UC mice. (**A**) ROS level in the mouse colonic tissues. (**B**) GSH level in the mouse colonic tissues. (**C**) MDA level in the mouse colonic tissues. (**D**) SOD activity in the mouse colonic tissues. (**E**) GSH-Px activity in the mouse colonic tissues. (**F**) T-AOC in the mouse colonic tissues. Data are expressed as mean ± SD (*n* = 3 of 10 mice). ^#^ *p* < 0.05, ^##^ *p* < 0.01, ^###^ *p* < 0.001 versus WT-control; * *p* < 0.05, ** *p* < 0.01 versus WT-DSS; ^&^ *p* < 0.05, ^&&^ *p* < 0.01, and ^&&&^ *p* < 0.001 versus SIRT3 KO-control.

Further experiments were conducted to assess the impact of SIRT3 KO on the antioxidative stress effect of CIM in the colonic tissues of UC mice. As measured by commercial assay kits, DSS induction elevated the ROS levels (Figure 11A), reduced the GSH concentrations (Figure 11B), increased the MDA levels (Figure 11C), impaired the enzymatic activities of SOD and GSH-PX (Figure 11D,E), and decreased the T-AOC levels (Figure 11F) in the colonic tissues of both WT and SIRT3 KO mice. Importantly, CIM treatment markedly suppressed ROS production, increased GSH concentrations, reduced MDA accumulation, enhanced SOD and GSH-PX enzymatic activities, and elevated T-AOC levels in the colonic tissues of WT mice. In contrast, no significant modifications in the aforementioned oxidative stress-related biomarkers were detected in SIRT3 KO mice after CIM administration (Figure 11A–F). Collectively, these findings demonstrated that SIRT3 knockout abolished the antioxidative stress effect of CIM in the colonic tissues of UC mice.

### 3.11. SIRT3 Knockout Weakened CIM-Induced Nrf2 Nuclear Translocation in the Colonic Tissues of UC Mice

**Figure 12:**
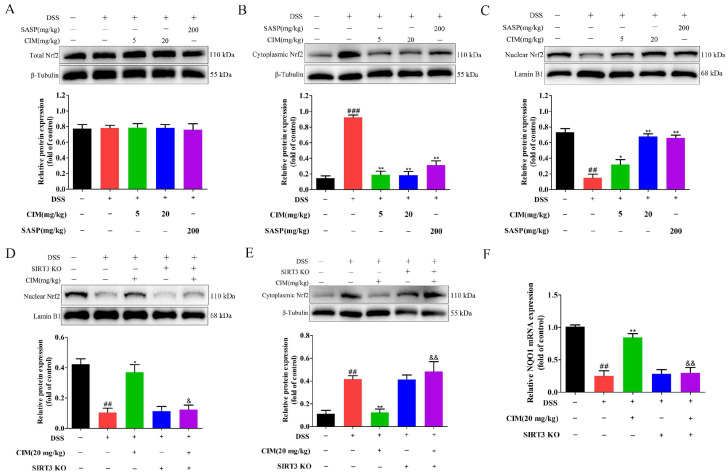
SIRT3 knockout abrogated the enhancement effect of CIM on the nuclear translocation of Nrf2 in the colonic tissues of UC mice. (**A**) Expression and quantification of total Nrf2 protein in mouse colonic tissues. (**B**) Expression and quantification of cytoplasmic Nrf2 protein in mouse colonic tissues. (**C**) Expression and quantification of nuclear Nrf2 protein in mouse colonic tissues. (**D**) Expression and quantification of nuclear Nrf2 protein in mouse colonic tissues. (**E**) Expression and quantification of cytoplasmic Nrf2 protein in mouse colonic tissues. (**F**) Expression of NQO1 mRNA in the mouse colonic tissues. Data are expressed as mean ± SD (*n* = 3 of 10 mice). ^##^ *p* < 0.01, ^###^ *p* < 0.001 versus control; * *p* < 0.05, ** *p* < 0.01 versus DSS; ^&^ *p* < 0.05, ^&&^ *p* < 0.01 versus DSS+CIM(20 mg/kg).

Nrf2 is a pivotal transcription factor that orchestrates cellular oxidative stress homeostasis [22]. However, whether Nrf2 mediates the regulatory effect of CIM on oxidative stress under the pathological conditions of UC remains unclear. To address this question, we further detected the influence of CIM on Nrf2 protein expression. The results revealed that CIM exerted no significant effect on the total Nrf2 protein level in the colonic tissues of mice (Figure 12A). Notably, DSS induction markedly upregulated cytoplasmic Nrf2 protein expression (Figure 12B) while downregulating nuclear Nrf2 protein expression (Figure 12C). Conversely, the CIM treatment decreased cytoplasmic Nrf2 accumulation and promoted nuclear translocation of Nrf2 in UC mice (Figure 12B,C). To further investigate whether SIRT3 is required for CIM-induced Nrf2 nuclear translocation, SIRT3 KO mice were employed. Compared with the control group, SIRT3 knockout markedly attenuated the effects of CIM, showing that both the CIM-mediated increase in nuclear Nrf2 accumulation (Figure 12D) and the reduction in cytoplasmic Nrf2 (Figure 12E) were significantly abolished. In addition, SIRT3 knockout also markedly attenuated the promotion effect of CIM on the mRNA expression of NQO1 in the colonic tissues of UC mice (Figure 12F). Collectively, these findings indicated that SIRT3 deficiency impaired the enhancement effect of CIM on Nrf2 nuclear translocation in the colonic tissues of UC mice.

## 4. Discussion

In the present study, we confirmed through in vivo experiments that CIM exerted a potent anti-UC effect, and its action was associated with the protection of intestinal mucosal barrier integrity, inhibition of colonic inflammation, and attenuation of oxidative stress. Most importantly, the pharmacological effects of CIM described above are mediated by the SIRT3 protein. Notably, the administration of CIM did not induce systemic toxicity in mice, indicating that CIM had excellent safety profiles for use. To our knowledge, there are few reports that evaluated the function of CIM in the treatment of DSS-induced UC via the suppression of oxidative stress and inflammation by regulating SIRT3.

Colonic inflammation is closely related to the progression of UC [23]. In UC, colonic inflammation is overactivated, accompanied by massive infiltration of inflammatory cells and excessive secretion of inflammatory cytokines, which destroy the intestinal inflammatory homeostasis. Persistent inflammatory injury without effective intervention will further aggravate the pathological process of UC and form a vicious cycle [24]. Therefore, inhibiting colonic inflammation can effectively alleviate the progression of UC. Previous studies have found that CIM exhibits a potent anti-inflammatory effect. A study by Hu et al. demonstrated that in a mouse model of airway inflammation induced by polyinosinic–polycytidylic acid (poly I:C), CIM treatment inhibited the levels of inflammatory cytokines and chemokines in bronchoalveolar lavage fluid and suppressed the infiltration of neutrophils into the lungs, thereby exerting an anti-airway inflammatory effect [18]. In addition, other studies have shown that CIM can reduce the phosphorylation level of IκB-α and decrease its degradation, thereby preventing the translocation of NF-κB p65 to the nucleus. This directly inhibits the expression of downstream inflammation-related genes, exerting an anti-inflammatory effect [17]. In the present study, CIM effectively restored the inflammatory balance in serum by inhibiting the levels of serum pro-inflammatory cytokines and increasing the levels of anti-inflammatory cytokines. Furthermore, CIM also suppressed the mRNA expression of inflammation-related proteins *COX-2*, *iNOS* and *TNF-α* in colon tissue, demonstrating a strong anti-colitic effect. This finding is consistent with the results of previous studies.

Oxidative stress is a key driver of UC pathogenesis [25]. UC progression causes intestinal epithelial injury and excessive ROS production, which in turn damage intestinal tissues. Moreover, elevated ROS downregulates tight junction proteins (e.g., ZO-1, Occludin), increasing intestinal epithelial permeability and further compromising the integrity of the intestinal barrier [26]. Therefore, inhibiting intestinal oxidative stress is a promising therapeutic strategy for UC. The present study demonstrated that CIM exerted a robust antioxidative stress effect by increasing the enzymatic activities, decreasing the levels of ROS and MDA, and elevating GSH levels as well as T-AOC, highlighting that CIM is a promising compound with anti-oxidative stress activity. Mechanistically, our results revealed that CIM did not alter the total Nrf2 protein abundance, but selectively promoted robust Nrf2 nuclear translocation, thereby transcriptionally activating downstream antioxidant effector pathways to scavenge reactive oxygen species, suppress oxidative toxicity, and restore damaged mucosal homeostasis. These findings highlight that modulating Nrf2 subcellular localization, rather than bulk protein expression, constitutes the core regulatory mode of CIM-mediated antioxidant defense. Further loss-of-function validation via SIRT3 knockdown revealed that genetic ablation of SIRT3 markedly abrogated CIM-induced Nrf2 nuclear import and its subsequent protective effects against colonic injury and oxidative stress. SIRT3, as a pivotal mitochondrial deacetylase, tightly governs cellular redox sensing and protein stability modification. Our observations further corroborate that SIRT3 acts as an indispensable upstream molecular switch to license Nrf2 nuclear activation, and the anti-UC pharmacological efficacy of CIM is strictly dependent on the intact SIRT3-Nrf2 signaling axis to maintain intestinal redox equilibrium.

Previous studies have shown that abnormal SIRT3 expression was closely linked to UC pathogenesis and serves as a key therapeutic target [27]. Under physiological conditions, SIRT3 modulates mitochondrial energy metabolism and repair, maintains mitochondrial homeostasis, and regulates cell survival and senescence. Downregulation of SIRT3 contributes to the progression of multiple diseases, including UC [28,29], whereas upregulation of SIRT3 can effectively alleviate the disease severity. Chen et al [27]. demonstrated that SIRT3 expression in the colonic tissues of UC patients was significantly lower than that in healthy controls. Additionally, in a DSS-induced UC mouse model, decreased SIRT3 expression was accompanied by shortened colonic length and increased DAI scores. Treatment with honokiol effectively upregulated SIRT3 protein expression in colonic tissues, thereby restoring colonic length, reducing DAI scores, and exerting anti-UC effects. Consequently, honokiol is also regarded as an agonist of SIRT3. Wang et al [16]. reported that active vitamin D_3_ could increase SIRT3 protein expression in the colon, reduce acetylated SOD2 levels, and subsequently decrease mitochondrial ROS production. This process inhibits ferroptosis in colonic tissues, thereby exerting anti-UC effects. In the present study, CIM could markedly upregulate the protein expression of SIRT3 in colon tissues, whereas it had no obvious effect on the enzymatic activity of SIRT3. Further molecular validation confirmed that CIM could directly bind to SIRT3 protein, suggesting that the direct interaction between CIM and SIRT3 contributed to the stabilization or upregulation of SIRT3 protein, rather than affecting its catalytic activity. Notably, knockout of SIRT3 significantly attenuated the anti-UC efficacy of CIM, indicating that SIRT3 served as a key target for CIM against UC. Given that SIRT3 is a key regulator of mitochondrial homeostasis and oxidative stress responses, its upregulation by CIM would logically enhance mitochondrial function, reduce ROS accumulation, and suppress intestinal inflammation. Consistently, SIRT3 knockout significantly abrogated the protective effects of CIM in UC models, confirming that SIRT3 is indispensable for a CIM-mediated anti-UC effect.

It is widely reported that SIRT1 mainly functions in colitis by regulating nuclear inflammatory signaling pathways [30]. Unlike SIRT1, SIRT3 is a key mitochondrial deacetylase that plays an essential role in maintaining mitochondrial function, energy metabolism, and redox homeostasis. In the present study, CIM showed no obvious effect on *SIRT1* mRNA expression, but markedly restored *SIRT3* mRNA expression, which further contributed to the improvement of mitochondrial redox balance, oxidative stress, and mitochondrial function in colonic tissue. Therefore, we reasonably proposed that SIRT3, rather than SIRT1, serves as a key molecule mediating the protective effect of CIM, especially via regulating mitochondrial redox homeostasis. This observation explains why the DSS-induced murine model closely mimics the core pathological features of intestinal mucosal injury and inflammatory infiltration observed in patients with UC. More importantly, the specific upregulation of *SIRT3* mRNA by CIM (with no impact on *SIRT1* or *SIRT6*) provides a critical breakthrough for elucidating the mechanism underlying CIM-mediated intervention in UC.

As a cycloartane-type triterpenoid glycoside, CIM possesses a typical polycyclic skeleton and multiple hydroxyl groups, which are crucial structural features for scavenging ROS, stabilizing mitochondrial membrane potential, and inhibiting oxidative stress. These functional groups contribute directly to its antioxidant capacity and mitochondrial protective effects. In addition, the triterpenoid core structure of cimigenoside is closely associated with the inhibition of pro-inflammatory signaling pathways (such as NF-κB and MAPK), thereby reducing the release of inflammatory cytokines and alleviating intestinal inflammation. The sugar moiety may further improve its solubility and bioavailability, facilitating its distribution and biological function in vivo, highlighting how its characteristic chemical structure supports the antioxidant and anti-inflammatory effects observed in this study. Notably, several cycloartane-type triterpenoids have been reported to exhibit potent anti-UC activities. Among them, astragalosides represent the most extensively investigated and best-evidenced class for the treatment of UC. Studies have demonstrated that astragaloside IV exerts remarkable anti-UC effects, mainly through the following mechanisms: (1) blocking the PI3K/AKT signaling pathway, thereby improving intestinal epithelial barrier function [31]; (2) remodeling the Th17/Treg balance and inhibiting the Notch pathway, consequently alleviating oxidative stress [32]; and (3) regulating the gut microbiota by increasing beneficial bacteria and reducing pathogenic bacteria [33]. Therefore, we hypothesize that CIM, which is also a cycloartane-type triterpenoid, may exert its anti-UC effects by regulating the above-mentioned mechanisms mediated by the SIRT3 protein.

In the present study, SASP was selected as the positive control drug because it is a well-recognized and widely used positive agent in this field, with confirmed efficacy and good comparability. Notably, our results showed that low-dose CIM could achieve comparable or even better effects than SASP in several key indicators, such as ZO-1 and Occludin expression in colonic tissues, the serum IL-6 levels, GSH levels, and SIRT3 mRNA and protein expression, suggesting that CIM exerts favorable efficacy, even at a relatively low dose. This indicates that CIM may have the advantages of a lower effective dose, higher potential safety, and broader clinical application prospects. Of note, a previous study has demonstrated that γ-secretase inhibitors can exacerbate colitis by altering the intestinal epithelial layer [34]. Given that CIM has been reported to inhibit γ-secretase, we speculate that prolonged administration of this compound may carry a potential risk of inducing colitis [35]. In addition, although the DSS-induced colitis model reliably recapitulates acute epithelial damage and redox imbalance, it cannot fully reflect the complex chronic immune dysregulation observed in clinical ulcerative colitis. Therefore, we acknowledge that the present study has inherent limitations regarding pathological translational extrapolation. Further validation using chronic colitis models and human biopsy specimens will be essential before clinical application, and such translational verification is currently prioritized in our ongoing research.

## 5. Conclusions

In conclusion, the present study demonstrates that CIM directly binds to SIRT3 and upregulates its protein expression, which in turn inhibits colonic inflammation and oxidative stress and restores the intestinal mucosal barrier’s integrity, thereby exerting anti-UC effects. These results highlight SIRT3 as a core mediator linking CIM’s direct binding to its therapeutic effects, providing novel insights into the pharmacological regulation of SIRT3 and laying a foundation for developing CIM-based strategies targeting SIRT3 for UC treatment. CBN represents a promising candidate compound for the treatment of UC.

## Data Availability

The original contributions presented in this study are included in the article/Supplementary Material. Further inquiries can be directed to the corresponding authors.

## Supplementary Materials

The following supporting information can be downloaded at: https://www.mdpi.com/article/10.3390/antiox15040428/s1.
