# Supplementary material for: Cimigenoside Attenuates Ulcerative Colitis by Inhibiting Oxidative Stress and Inflammation via Sirtuin 3 Enhancement in Mice
Source: Antioxidants (Basel). 2026 Mar 28;15(4):428. doi: 10.3390/antiox15040428 (PMC13113098; doi:10.3390/antiox15040428)
Supplement: Supplementary file 1 [file antioxidants-15-00428-s001.zip › antioxidants-4142879-supplementary.pdf]

Product Specification

Small Molecule Compounds

产品信息 / Product information

产品货号 / Cat No.	IYT1771
CAS NO.	27994-11-2
中文名称 / Chinese Name	升麻环氧醇苷
英文名称 / English Name	Cimigenoside
保存 / Storage	Powder:2-8°C，2 years;Insolvent(母液):-20°C，6 months;-80°C，1 year

化学属性 / Chemical properties

分子式 / Formula	$C_{35}H_{56}O_9$
分子量 / Molecular Wt.	620.81
外观 / Appearance	White to off-white Solid
纯度 / Purity	≥98%

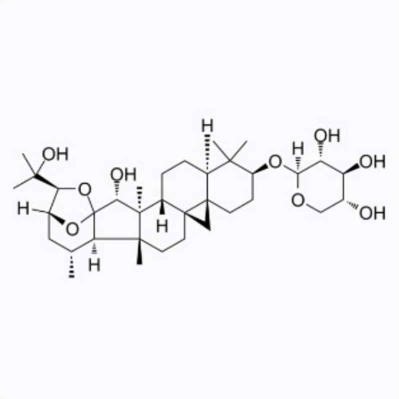

生物活性 / Biological activity

背景说明 / Description	据报道，Cimigenoside 是一种 γ-分泌酶抑制剂，通过 γ-分泌酶/Notch 轴抑制人类乳腺癌细胞的增殖或转移。
靶点 / Target	γ-secretase
通路 / Pathway	Stem Cells;Neuronal Signaling

溶解性 / Solubility

In Vitro:

Soluble in Water ≥10mg/mL

"≥" means soluble, but saturation unknown.

* 配成工作液后，建议立即使用，尽快用完。

* 产品为非无菌包装，请根据实验需求提前做好预处理。如需配成储备液，建议分装保存，避免反复冻融造成的产品失效。

* The product is non-sterile packaging, please do pre-treatment in advance according to experimental needs. If it is necessary to prepare a stock solution, it is recommended to store in aliquots to avoid product failure caused by repeated freezing and thawing.

溶液制备参考 / Solution preparation reference

Mass / Solvent / Concentration	1mg	5mg	10mg
1mM	1.6108 mL	8.0540 mL	16.1080 mL
5mM	0.3222 mL	1.6108 mL	3.2216 mL
10mM	0.1611 mL	0.8054 mL	1.6108 mL

相关文献 / References

（See more information on https://www.solarbio.com）

（若有投稿意向，欢迎关注我公司奖学金计划 https://www.solarbio.com/articleList/articleInfo?id=1178）

（Welcome to follow our company scholarship program https://www.solarbio.com）
